# 90-90-90 – Charting a steady course to end the paediatric HIV epidemic

**DOI:** 10.7448/IAS.18.7.20296

**Published:** 2015-12-02

**Authors:** Elaine J Abrams, Susan Strasser

**Affiliations:** 1 ICAP, Mailman School of Public Health, Columbia University, New York, NY, USA; 2 Department of Pediatrics, College of Physicians & Surgeons, Columbia University, New York, NY, USA

**Keywords:** paediatric HIV, prevention of mother-to-child HIV transmission, antiretroviral treatment, antiretroviral adherence

## Abstract

**Introduction:**

The new “90-90-90” UNAIDS agenda proposes that 90% of all people living with HIV will know their HIV status, 90% of all people with diagnosed HIV infection will receive sustained antiretroviral therapy and 90% of all people receiving antiretroviral therapy will have viral suppression by 2020. By focusing on children, the global community is in the unique position of realizing an end to the paediatric HIV epidemic.

**Discussion:**

Despite vast scientific advances in the prevention and treatment of paediatric HIV infection over the last two decades, in 2014 there were an estimated 220,000 new paediatric infections attributed to mother-to-child HIV transmission (MTCT) and 150,000 HIV-related paediatric deaths. Furthermore, adolescents remain at particularly high risk for acquisition of new HIV infections, and HIV/AIDS remains the second leading cause of death in this age group. Among the estimated 2.6 million children less than 15 years of age living with HIV infection, only 32% were receiving life-saving antiretroviral treatment. After decades of languishing, good progress is now being made to prevent MTCT. Unfortunately, efforts to scale up HIV treatment services have been less robust for children and adolescents compared with adult populations. These discrepancies reflect substantial gaps in essential services and numerous missed opportunities to prevent HIV transmission and provide effective life-saving antiretroviral treatment to children, adolescents and families. The road to an AIDS-free generation will require bridging the gaps in HIV services and addressing the particular needs of children across the developmental spectrum from infancy through adolescence. To reach the ambitious new targets, innovations and service improvements will need to be rapidly escalated at each step along the prevention-treatment cascade.

**Conclusions:**

Charting a successful course to reach the 90-90-90 targets will require sustained political and financial commitment as well as the rapid implementation of a broad set of systematic improvements in service delivery. The prospect of a world where HIV no longer threatens the lives of infants, children and adolescents may finally be within reach.

## Introduction

In October 2014, in a bold effort to accelerate an end to the AIDS epidemic, UNAIDS proposed ambitious new targets to accelerate the HIV treatment scale-up in low- and middle-income countries [1]. The targets, described as “90-90-90,” propose that 90% of all people living with HIV will know their HIV status, 90% of all people with diagnosed HIV infection will receive sustained antiretroviral therapy and 90% of all people receiving antiretroviral therapy will have viral suppression by 2020 [1]. It is posited that achievement of these 90-90-90 targets in the next decade has the potential to transform and likely end the AIDS epidemic by 2030. Unimaginable a decade ago, the prospect of an AIDS-free world may finally be within reach. Charting a successful course to an AIDS-free world, however, will require sustained political and financial commitment as well as a series of systematic improvements in service delivery. This is particularly the case as we apply the 90-90-90 paradigm to paediatrics which would require identifying 3.7 million infants, children and adolescents with HIV infection, treating 3.3 million and achieving viral suppression among 3 million within the next four years.

Remarkable achievements connote the history of the antiretroviral treatment (ART) scale-up in low- and middle-income countries with more than 13.5 million individuals initiated on treatment and a slow but steady restoration of health and stability to many communities devastated by HIV infection [2]. Children have also benefited from global efforts to prevent and treat paediatric HIV infection but successes, particularly in efforts to scale up treatment, have been less robust compared with adult populations. Despite substantial scientific advances in the prevention and treatment of paediatric HIV infection, in 2014 there were 220,000 new paediatric infections attributed to mother-to-child HIV transmission (MTCT), and in 2013, close to a quarter of a million new HIV infections reported in adolescents 15–19 years of age [3, 4]. Among the estimated 2.6 million children less than 15 years of age living with HIV infection, only 32% were receiving life-saving ART. Furthermore, in 2013, 210,000 children and 120,000 adolescents died because of HIV/AIDS [4, 5]. HIV is now the second leading cause of death among adolescents, second only to road traffic injuries. It is estimated that 32% of adults living with HIV are virally suppressed but global estimates for children are not available [6]. Reported viral suppression rates among children on ART therapy in low- and middle-income countries range from 50 to 90%, varying by regimen, treatment duration, geographic location, age and developmental stage [7–10]. Overall, these statistics not only reflect notable accomplishments in comparison to years past, but also underscore the multiple gaps in essential services and numerous missed opportunities to prevent, treat and successfully manage HIV infection. The road to an AIDS-free generation will require bridging the gaps in HIV services and addressing the particular needs of children across the developmental spectrum from infancy through adolescence.

In well-resourced countries where scientific discoveries have been rapidly translated into policy and clinical practice, new paediatric HIV infections are increasingly rare, and most children living with HIV have aged into adolescence and adulthood. Consistent and widespread access to comprehensive antenatal care coupled with routine HIV testing and combination antiretroviral therapy for pregnant women has resulted in MTCT rates of less than one percent in many settings [11, 12]. At the same time, the availability of an ever-increasing and improving armamentarium of highly efficacious, virally suppressive ARTs has transformed perinatal HIV infection into a chronic, treatable disease of adolescence and adulthood. In the United Kingdom and Ireland, in 2011, the average age of 1131 children followed in the Collaborative HIV Paediatric Study was 13.2 years, with approximately 1/3 of children over 15 years of age [13]. Similarly, in 2011 in New York City, an epicentre of the paediatric HIV epidemic in the United States, among 2449 children with perinatal HIV, 11% were reported to be older than 24 years of age with 76% between the ages of 13 and 24 years [14].

While the population of children with perinatally acquired HIV is ageing in the global north, this is juxtaposed against persistently high transmission and mortality rates amongst affected paediatric populations in sub-Saharan Africa. What will it take to further accelerate prevention and treatment efforts in sub-Saharan Africa and other resourced constrained settings to achieve successes similar to those in the global north? Unfortunately, ART was late to arrive in sub-Saharan Africa where more than 90% of children with HIV infection live and, for many years, efforts to prevent perinatal infections were hampered by repeated attempts to find quick, easy and inexpensive approaches rather than building an enabling platform of HIV services to deliver efficacious antiretroviral therapy to pregnant women to prevent MTCT and treat maternal HIV infection [15, 16]. The road has been bumpy and mired by mixed messages on breastfeeding and frequent shifts in prevention of mother-to-child transmission (PMTCT) drug regimens and approaches. More recently, important incremental improvements and innovations across the paediatric prevention–treatment cascade have resulted in better outcomes – fewer new infections, and decreased morbidity and mortality for those with established disease. To reach the ambitious new targets, however, innovations and service improvements will need to be rapidly escalated at each step along the cascade.

## Discussion

### Preventing new paediatric HIV infections

First proposed as a solution to the poorly functioning PMTCT programme in Malawi, the Option B+ approach was hotly debated [17]. It was believed, on one hand, that Option B+ would pave the road to elimination of new paediatric infections by initiating lifelong treatment for all pregnant and breastfeeding women, maternal health would be protected and transmission would be prevented during current and future pregnancies, and to uninfected partners [18]. On the other hand, there was deep concern that Option B+ would result in costly, unnecessary treatment for millions of women and dangerous drug exposures for their babies. Four years later, the Option B+ approach has been endorsed by the majority of high HIV burden countries in sub-Saharan Africa, and on balance, early field experience suggests that it has leapfrogged over other approaches to increase ART uptake among pregnant and breastfeeding women and reduced loss along the first steps of the PMTCT cascade from HIV diagnosis to treatment initiation [19, 20]. This approach, though initially engendering scepticism, has charted a course for potential systematic improvements in care. Several countries have reported dramatic increases in the number of HIV-positive pregnant and breastfeeding women starting ART [20]. While this is excellent news, early reports also suggest that retention on treatment is less than optimal and substantial numbers of women on Option B+ do not remain engaged in care [20–23]. It appears that some of the early losses in the PMTCT cascade, between diagnosis and treatment initiation, may simply have been postponed rather than prevented [23].

A wide array of individual, structural and health systems factors contribute to whether a woman and her baby can and will stay in care [17–20,22–30]. Many of these factors overlap with those described by non-pregnant adults as reasons for non-retention including high out-of-pocket costs, long travel times, inconvenient clinic hours, poor treatment by health workers and self-transfer to other health facilities. Each of these factors, either individually or collectively, threatens achievement of elimination goals. Option B+ has been generally interposed onto an already fragile maternal-child health (MCH) structure ill-equipped to provide comprehensive treatment services during pregnancy and especially during the postpartum period. In addition, social and cultural influences affect health seeking behaviour and health service use during this time period. In many settings, pregnant women migrate to family homes for delivery and the early postpartum period, and some may leave their babies with families when they return to work. In some parts of the world, access to and uptake of antenatal care is low, and large numbers of women are unable to learn their HIV status or obtain HIV care in a timely way. Finally, despite an explosion of electronic health information systems and mobile health technologies globally, penetration in MCH services has been poor, and the paper-based medical record and register-based systems remain the norm [31]. Different service delivery models and nimble, responsive and adaptive health platforms are needed to expand access to care and improve service delivery and retention along the PMTCT cascade.

Attention urgently needs to shift downstream in the PMTCT cascade, from getting women on ART to engaging them in lifelong care, and to augmenting the health systems now responsible for providing these services. This can only be done by applying successful interventions already in place in services where non-pregnant adults traditionally receive ART, but also by understanding and addressing the particular needs of women and their children during this period of time [32]. In addition to reinforcing the MCH platform to ensure that PMTCT services can be delivered, innovations both small (organized appointment systems, SMS reminders, extended prescriptions, mother–baby registers) and large (integrated mother–child postnatal follow-up, community-based ART, engaging partners and communities, home-based HIV testing, multi-drug infant prophylaxis regimens) will be needed to identify, engage and retain all women and infants at risk and minimize loss and enhance efficiency across the PMTCT cascade. Without implementing improvements in how services are delivered, efforts to prevent new infections among infants will be compromised.

### Identifying and engaging children with HIV infection

Weaknesses in PMTCT programming have been particularly evident on the infant side of the cascade where early infant diagnosis (EID) and early treatment are critical to prevent rapid disease progression and reduce the high mortality rates well documented in untreated infants and young children [33]. In 2013, only 42% of HIV-exposed infants in the 21 priority countries received an early diagnostic test in the first two months of life to determine infection status [3]. Performance across subsequent steps in the cascade wanes dramatically with few HIV-exposed children remaining in care throughout the period of breastfeeding and MTCT risk. Furthermore, having a positive EID test has not directly translated into early ART initiation due to long delays from the time of taking the test to getting results to the family, preparing for and finally starting treatment [34]. Improvements in how PMTCT is delivered, as described above, are likely to have the greatest impact on infant outcomes first and foremost by reducing the number of babies acquiring HIV infection, but equally importantly, by shifting the HIV care paradigm to one that facilitates long-term, continuous tracking and engagement of mothers and their infants and by providing a platform on which to rapidly deliver diagnostic, prevention and treatment services. A variety of other innovations have been demonstrated to expedite various steps along the EID cascade such as: the use of SMS printers to return test results to health facilities quickly and systematically, engaging peer and community workers to help patients remain in care and reduce stigma, computer-based tracking systems, and expanding EID testing to additional access points beyond PMTCT programmes [35, 36]. Other approaches of great interest include the addition of an EID test at birth and introducing point-of-care (POC) diagnostics at the clinic level [37, 38]. While each has the potential to improve early identification and treatment of HIV-infected infants, it will be important to carefully consider the consequences of adding more demands onto the fragile PMTCT-EID infrastructure currently in place. Health workers juggle numerous competing demands, and facilities are often under-resourced and poorly staffed. It is of note that many country programmes are still challenged to provide the basics, such as an uninterrupted supply of rapid HIV tests to PMTCT and other clinical services.

Challenges in case finding have not been limited to infants [39]. While global estimates for the number of children living with HIV have recently been reduced from 3.2 million to 2.6 million, we find that fewer than half of all children living with HIV are diagnosed and actively engaged in care [6]. However, when concerted efforts are made to test children, not surprisingly, large numbers of HIV-infected infants, children and adolescents are identified. HIV programmes, however, have generally failed to implement routine HIV testing for children [40, 41]. Intentional and routine testing, even in high burden areas, is the exception rather than the norm. Through provider-initiated testing and counselling (PITC), inpatient wards and malnutrition, tuberculosis and urgent care clinics have been demonstrated to be high-yield venues to identify HIV-infected children, but resources are rarely available to offer routine testing [42, 43]. Testing the children of adults with HIV should also improve paediatric case finding, but adults are often unable or unwilling to bring their children and families to the clinic. Health workers are also often reluctant to initiate the conversations and counselling necessary to promote paediatric testing. Ahmed *et al*. trained community health workers in Malawi to provide clinic- and home-based testing of paediatric contacts of adult index patients, demonstrating the value of family/household testing and the importance of community-based approaches to find HIV-infected children [36]. Similarly, by introducing routine PITC in primary care clinics in Zimbabwe, Ferrand *et al*. successfully identified adolescents with HIV infection, many with symptomatic disease and presumed perinatal infection [40, 44].

If we are to reach the first target, to ensure that the status of 90% of all positive infants, children and adolescents is known, there will need to be proper resourcing for and a rapid scale-up of routine testing in these high-yield settings, both in health centres and communities. This will require making HIV testing a routine part of health services for paediatric populations particularly in high prevalence settings, training health workers on the importance of and proper approaches to testing children, improving the supply chain for HIV test kits, addressing issues around consent for paediatric and adolescent testing (e.g. age and authority to provide consent), developing effective systems to link those testing positive to HIV care and collecting data disaggregated by age to ensure better programme monitoring. Recent guidance from the United States President's Emergency Fund for AIDS Relief (PEPFAR) summarizes key strategies for identifying and linking infants, children and adolescents to HIV care and are included in Table 1 [45].

**Table 1:** Strategies to improve paediatric case finding and antiretroviral treatment^a^

	Area	Description
Cross-cutting strategies	Health work force	Increase number and capacity of health workers to provide paediatric and adolescent services; task shifting and sharing; engagement of community and lay worker cadres; health worker training; mentoring and continuing education
	Service delivery	Decentralization; integration of services including youth-friendly services and sexual and reproductive health; community, family and home-based testing, care and treatment; reduce out-of-pocket expenses; appointment systems with active tracking and follow-up including SMS and telephonic reminders
	Supply chain	Improve supply chain management of paediatric antiretroviral medications and essential commodities including HIV test kits and early infant diagnosis materials
	Monitoring and evaluation	Collect age-disaggregated testing and treatment data; document linkages between mothers and infants, testing and enrolment, transfers between facilities; improve data quality; develop approaches to routinely measure key outcomes including mother-to-child transmission rates, viral suppression, HIV drug resistance
Strategies to improve HIV testing	Early infant diagnosis	Point-of-care diagnostics; birth testing in addition to routine testing at six weeks of age; expand testing to access points outside of PMTCT programmes; centralized specimen transport schemes; SMS printers to return results to facilities
	Testing of older children and adolescents^a^	Test all children and adolescents of adults receiving HIV services through facility or home-based testing; admitted to inpatient paediatric wards; attending TB clinics, malnutrition and urgent care services; receiving orphan and vulnerable children (OVC) services
		Test mothers or infants attending immunization or under-5 clinics to identify HIV-exposed infants in high prevalence settings (>5%),
Strategies to improve HIV treatment		Universal ART for all infants, children and adolescents; remove CD4 and clinical criteria for ART initiation
		Improve drug regimens and formulations: fixed-dose formulations; dispersibles; scored adult tablets; weight band guided dosing
		Expedite development and early access for new drugs and drug combinations with improved efficacy and resistance profiles for children
		Integrated age-appropriate psychosocial and behavioural services including disclosure, adherence and sexual and reproductive health

a Adapted from “Strategies for Identifying and Linking HIV-Infected Infants, Children, and Adolescents to HIV Care and Treatment,” www.pepfar.gov/documents/organization/244347.pdf.

### Treatment for children with HIV infection

An estimated 832,000 children less than 15 years of age were reported to be receiving treatment in low- and middle-income countries in 2014 [6]. While increasing numbers of children are initiated on treatment annually, the number will need to be tripled to meet the 90-90-90 targets. Treatment coverage among children varies by geographic region, by country and, within countries, by region and district [32]. Some countries in sub-Saharan Africa, where the burden of HIV infection is greatest, have met with notable success, while others, particularly in West Africa, have seen more modest improvements in paediatric treatment. Additionally, children are still entering care late and initiating treatment at advanced stages of disease [46, 47]. Infants and adolescents have been particularly challenging to identify and initiate on treatment, contributing to high rates of mortality and suboptimal treatment outcomes in these very high risk groups [48–50].

The impediments to treatment in paediatric populations parallel those cited for testing including patient and system-level barriers. The complexity of paediatric treatment, including age and CD4-specific indications for initiation, weight-based dosing, and the need for different regimens and formulations across age groups, contrast distinctly with the simplicity of the uniform “one pill once daily” approach to adult ART. Limited health worker capacity and reported high levels of discomfort treating children have often been cited as contributing to the limited ART coverage among children.

Echoing the Option B+ debates, the proposal to treat all children, independent of age or severity of disease, has engendered heated discussion. Those in favour of universal treatment cite CD4 testing as a barrier to ART initiation and a step along the cascade with high risk for loss [51]. Retention among those not eligible for treatment (pre-ART) is substantially worse compared with children on therapy and rather than providing a venue for close monitoring, many children are lost to care, often returning late with more advanced disease [52, 53]. In addition, an emerging body of evidence suggests that some health outcomes such as immune restoration may be better when treatment is started earlier among healthier children [54, 55]. Several countries in sub-Saharan Africa, such as Zambia and Uganda, have already pushed ahead and endorsed treatment for all children under 15 years [56]. In light of findings from two adult trials demonstrating the benefit of ART in adults with high CD4 counts, the World Health Organization will recommend universal treatment for all individuals living with HIV [57, 58]. As these recommendations are taken up by national programmes, we can expect to see, similar to the B+ experience [19, 20], an uptick in the number of children on treatment in low- and middle-income countries.

In addition to simplifying ART eligibility, increasing health worker capacity to care for children through training, mentoring and sustained supervision and improving supply chain management of antiretrovirals will be essential to achieve the ambitious treatment targets. Furthermore, decentralization to primary care facilities and innovations in service delivery such as family-focused care; youth-friendly services; and community-, school- and home-based ART [59–61] are long overdue for paediatric populations. There is also a pressing need to engage with the behavioural and psychosocial issues affecting children, adolescents and families affected by HIV, particularly disclosure, mental health and, for adolescents, to ensure easy access to sexual and reproductive health services [62, 63].

## Achieving and maintaining viral suppression

Population-based estimates of viral suppression among paediatric populations are not available. In published reports, suppression rates vary from 40% to over 90%, by population, age, gender, drug regimen, calendar year, duration of ART, study design and frequency of measurement. Among 4803 children on ART in South Africa, in crude analyses at any time-point on treatment, 65.9% (95% CI 62.7–68.4%) of children receiving a community-based intervention and 55% (95% CI: 54.2–57%) in the standard of care arm were virally suppressed [7]. In a recent cross-sectional analysis of adults and children on ART in Swaziland, rates of suppression were reported at 71, 65, and 86% among <10, 10–19, and 20 years and older [8]. Achieving and maintaining viral suppression among adolescents has been particularly difficult across high- as well as low- and middle-income countries [64].

The most substantial threat to the third target has been, and continues to be, the inferior drugs and formulations available for treating children. Adult treatment has been revolutionized by new drug classes and formulations, with fixed-dose once-daily combinations of highly efficacious medications with improved toxicity profiles. Not yet widely available in resource-poor settings, many of these agents appear to be on track to enter the global market in the near future. These advances reflect consistent, aggressive efforts over the past decade to expand and improve treatment access for adults with HIV infection.

The efforts on the part of children have been less consistent and generally less successful. This is compounded by the biology of childhood, which implicitly makes paediatric drug development more complex and further limits the drug formulary for children, particularly in low- and middle-income countries. Physical growth and organ maturation directly impact drug metabolism, warranting study of dosing and safety across the age/weight spectrum of childhood and adolescence. Special formulations (granules, dispersibles and syrups) are needed to ensure proper dosing for infants and young children. Therefore, paediatric drug development is often decades behind that of adults, and an array of less optimal drugs remain the only options for children. Urgent measures are needed to expand and expedite paediatric drug development and to guarantee early access to the safest, most efficacious regimens. Long-acting formulations, currently in trial in adult populations, hold particular promise for adolescents, where the use of other long-acting formulations met with great success [65, 66]. In the interim, it remains necessary to assess innovative strategies to best employ currently available drugs and settle for less optimal and often more complex options to expand treatment access and prevent disease progression and mortality [67, 68]. Efforts to strengthen health systems as noted in the previous sections are also imperative not only to increase the number of children initiating treatment but also to improve the quality of care including providing effective adherence, monitoring and support, and maintaining an uninterrupted supply of essential medications.

Finally, achieving and maintaining viral suppression amongst 90% of children on treatment will require fully embracing the challenges of adherence to lifelong treatment as well as the complexity of paediatrics, recognizing and addressing the evolving emotional and psychosocial needs of children as they grow and develop. Children need to learn their HIV status when it is developmentally appropriate, and adolescents need to be equipped with the knowledge and skills to adhere and protect themselves and their partners as they age and mature. Emergency efforts to make these life-saving drugs available have, to date, left little room to consider the emotional wellbeing of these children and their families [69, 70]. Globally, HIV-infected children are often from vulnerable families affected by poverty, violence, limited health care and educational resources, and not infrequently, racism and discrimination. They often experience disruptions in caregiving due to a variety of factors including parental illness and death, and live in communities where HIV stigma is prevalent. Added to this list are the challenges of adhering to a daily medication regimen and grappling, when they are aware, with the knowledge of having a transmissible, potentially fatal infection. Adolescence is a particularly vulnerable time when normal neurologic and developmental changes increase the risk of acquisition of HIV infection and jeopardize retention and adherence among those with established disease [64, 71–73]. Expanding services and health worker capacity to address the psychosocial needs of children and adolescents living with HIV will be essential to fully realize the potential of the ART scale-up [74].

## Conclusions

In the history of the AIDS epidemic, targets have been enormously helpful to focus resources and attention on global inequities in treatment and other essential services. While they are oftentimes largely aspirational, the “3 by 5” initiative propelled the treatment scale-up in low- and middle-income countries [75]. For infants, children and adolescents with HIV, we find ourselves at a moment in time when political commitment and financial resources are aligned, opening a door to improving health outcomes for this special population [76]. The 90-90-90 campaign offers the prospect of a world where HIV no longer threatens the lives of infants, children and adolescents.
